# The effect of morning versus evening administration of empagliflozin on its pharmacokinetics and pharmacodynamics characteristics in healthy adults: a two-way crossover, non-randomised trial

**DOI:** 10.12688/f1000research.51114.1

**Published:** 2021-04-26

**Authors:** Rana M. ElDash, Mohamed A. Raslan, Sara M. Shaheen, Nagwa Ali Sabri

**Affiliations:** 1 Pharmacy Practice Department, Faculty of Pharmacy, Heliopolis University, Cairo, 11785, Egypt; 2 Quality Control, Drug Research Center, Cairo, Egypt; 3 Clinical Pharmacy Department, Faculty of Pharmacy, AinShms University, Cairo, 11566, Egypt

**Keywords:** Chronopharmacology, Empagliflozin 10 mg, Diabetes Mellitus, Bioequivalence, morning dose, evening dose, Pharmacokinetics, Pharmacodynamic, Circadian rhythm.

## Abstract

**Background**: Empagliflozin is an SGLT2 inhibitor approved for use in patients with diabetes mellitus type 2 (DMT2) with or without other cardiovascular disease. Empagliflozin is taken once daily without rationale on the optimal timing for administration. This study aimed ****to determine the chronopharmacological effects of morning vs evening administration of empagliflozin (10 mg) in healthy Egyptian adults, by investigating the pharmacokinetics and pharmacodynamics parameters of empagliflozin depending on the intake time.

**Methods: **An open label, sequential, two‐way crossover trial comprised of two periods with a washout period of 7 days. All participants received a single oral dose of empagliflozin (JARDIANCE ®; 10 mg film coated tablet) in the evening, and after a seven-day washout period, the morning. Pharmacokinetics parameters (primary endpoints: t _max_ (h), C _max_ (ng/ml), AUC _0-t_ (ng.h/ml); secondary endpoints: AUC _0 to ∞_(ng.h/ml)) were assessed. Method validation was done prior to injection in LC/MS/MS and samples were processed by Liquid-Liquid extraction. The pharmacodynamic profile (UGE _0-24_) was determined after method validation (glucose hexokinase method).

**Results: **T _max_ increased by 35% in the evening phase compared to the morning phase, while C _max_ decreased by -6.5% in the evening dose compared to the morning dose. Additionally, AUC _0 to ∞_ increased in the evening phase by 8.25% compared to the morning phase. The mean cumulative amount of glucose excreted (UGE ( _0-24_)) increased by 43% in the evening dose compared to the morning dose

**Conclusion**: ****Despite the difference in pharmacokinetics parameters between evening and morning doses, C _max_, AUC _0-t_, AUC _0-∞_, didn’t differ on the bioequivalence level. In addition, as UGE ( _0-24_) didn’t statistically differ, thus, we can conclude that there is no statistical significance between the morning and evening doses.

**Trial registration: **Clinal Trials.gov, ID: NCT03895229 (registered on 29 ^th^ March 2019).

## Introduction

Diabetes mellitus (DM) is one of the leading causes of death in the world. It has become one of the most critically medical and socially impacted diseases of the 21 ^st^ century. Moreover, it is considered an epidemic disease with very high proportion of cases discovered every year. According to the World Health Organization (WHO), the prevalence of DM worldwide almost doubled from 4.7% to 8.5% between 1980 and 2014, reaching approximately 422,000,000 of diabetic adults in 2014, particularly in low-and middle-income countries like Egypt ^1^. By 2040, around 642 million people will be diagnosed with DM ^2, 3^.

Chronopharmacology is the field of study concerned with the circadian rhythm of drugs regarding its pharmacokinetics/pharmacodynamics and pharmacological action; it also determines how the timing of the day may change the pharmacological action, pharmacokinetics and pharmacodynamics of the drug. Results of chronopharmacological studies are taken into practice under the umbrella of chronotherapy ^4^. In addition, chronopathology proposes that any disease, including DM, can happen as a result of disruption of biorhythms ^5, 6^.

The pathogenesis of DM involves different types of models, which explain various mechanisms of its pathogenetic development. For many years, the glucocentric approach has shaped the main theory of DM development. The new multigene concept shifted the theory of glucocentricity, and as a result, it added a new pathway for treating DM; many theories are presented which may open up our understanding on how circadian rhythm affects DM ^5^.

Circadian rhythm influences the kidney; the glomerular filtration rate (GFR) and renal plasma flow, together with tubular secretion and absorption, work promptly in the active phase and decrease activity in the inactive phase. These mechanisms are controlled by a circadian clock, which dominates multiple cellular functions, such as the transcription and translation of proteins, the addition of phosphorous or acetyl group, and even the ubiquitylation of protein (post transitional). Moreover, kidney functions (electrolyte excretion, urine volume and regulation of blood pressure) abide by circadian variation ^7, 8^.

These various circadian etiologies affecting the development of DM necessitate the study of how chronopharmacological studies on antihyperglycemic drugs can affect the outcome of the treatment. This is the aim of personalized medicine. However, there are only a few available chronopharmacological studies to date available for antihyperglycemic drugs ^5^.

In this study, we propose to investigate empagliflozin. Empagliflozin, which acts by inhibiting the sodium glucose co-transporter 2 (SGLT2) in the proximal tubules in the kidney, is indicated for two major isoforms (SGLT1 and SGLT2) in DM type 2, which are proposed for the sodium-glucose cotransporter (SGLT). SGLT2 is mainly expressed in the lumen of the small intestine and kidneys; SGLT2 takes part in the absorption/reabsorption of glucose induced by the sodium gradient across the cell membrane in the proximal tubules in the kidney ^9^. Empagliflozin shows high improvement in glucose metabolism and, hence, its homeostasis. When added to standard care, it demonstrates, along with its anti-glycemic effect, a decrease in the patients’ mortality rate attributed to the cardiovascular disease(s), hospitalization for heart failure, all-cause hospitalization, and all-cause mortality ^10^. As a result, in 2016, the FDA announced that empagliflozin is indicated for diabetic patients with heart failure ^11^. In addition, in 2021, a new drug application is being investigated for empagliflozin in reducing risk of mortality or hospitalization and preserve kidney function in diabetic and non-diabetic with heart failure ^12, 13^. Moreover, it has a positive impact on glycemic control and reductions in weight and normalization of circadian blood pressure rhythm (non-dipping) ^9^.

Empagliflozin (JARDIANCE ® 10 mg film coated tablet) is found in the market in two concentrations (10 or 25 mg), taken once daily with no rationale on the intake time between the morning versus the evening ^10, 11^.

Empagliflozin is swiftly absorbed after single escalating oral doses (0.5–800 mg) with a two phases decline. AUC _0-∞ _and C _max_ are directly proportional with increasing doses. Moreover, in a few studies administration of empagliflozin with food resulted in halting the absorption, and in addition, the total quantity of glucose excreted increased by increasing the doses; it inhibited 40% of reabsorbed glucose at the single daily doses ^2^. 

The timing of drug intake can affect the circadian rhythm. For example, the blood pressure lowering drug Fimasartan shows pronounced blood pressure lowering (readjusting the dipping pattern) at night compared to morning dose. This is similar to our study drug, empagliflozin, which shows a provisional effect in restoring circadian blood pressure also at night in patients suffering from an imbalance in dipping blood pressure rhythm ^14, 15^.

In addition, since empagliflozin works on the kidneys’ proximal tubules, therefore, by examining the effect of inhibiting SGLT2 in the proximal tubules from a circadian rhythm point of view, it can add a new perspective in studying how the circadian rhythm can affect the timing of drug administration in the kidney.

The aim of this study is to examine the chronopharmacological characteristics of empagliflozin (10 mg film coated tablet) by comparing the antihyperglycemic effect (morning vs evening administration) on healthy adults, and to examine the influence of circadian rhythm on empagliflozin. Therefore, we examined for the first time the effect of the day and night dosing on the circadian rhythm for an antihyperglycemic drug, which may aid improving the proper use of this drug.

## Methods

### Study design

**Figure 1.**
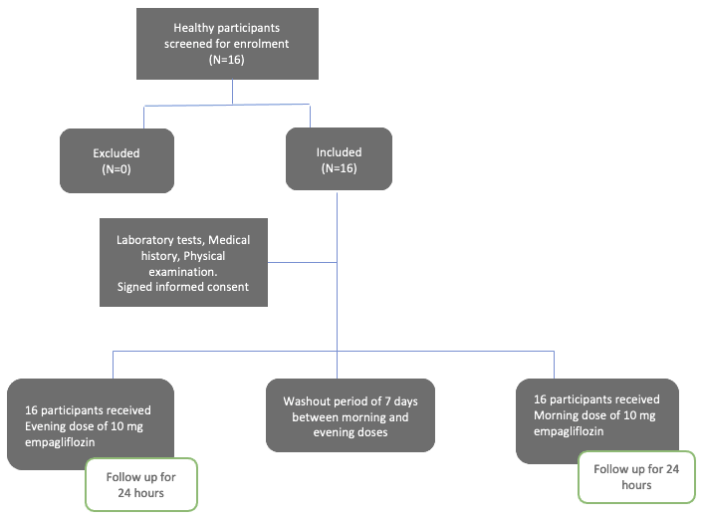
Flow chart of the study design of the effect of morning vs evening administration of empagliflozin (Jardiance® 10mg film coated tablet; manufactured by Boehringer Ingelheim Pharma Gmbh & Co, Germany) on its pharmacokinetics and pharmacodynamics.

This open label, sequential trial consisted of two periods with a seven-day washout period and took place between 2 ^nd^ and 10 ^th^ October 2018. Participants were selected 21–27 days before the trial between the 3 ^rd^ of September to 26 ^th^ of September 2018. Figure 1 includes the trial study design.

### Ethical statement

The trial was conducted at the Drug Research Center, Cairo, Egypt in compliance with Good Clinical Practice and in accordance with the International Conference of Harmonization (ICH) guidance on general considerations for clinical trials and the Declaration of Helsinki. The trial was approved by the Ethics Committee (IRB) of the Faculty of Pharmacy of Ain Shams University (no.203) and the Drug Research Center. The trial was registered in ClinicalTrials.gov (ID: NCT03895229) on 2 ^nd^ April 2019. The trial was registered after the trial had been completed due to an administrative error. The authors confirm that all ongoing and related trials for this drug/intervention are registered.

Each participant provided written informed ethic to participate in the trial prior to enrolment.

### Participants

Participants were selected 21–27 days before the trial and were recruited according to eligibility criteria from the drug research center volunteers’ database and volunteer referrals. In addition, dietary regimens (meals) were supplied in accordance with the FDA and Food and Nutrition Board, and all participants received the standard total caloric intake/day of fats (20–35%), carbohydrates (45–65 %) and protein (10–35%) ^16^.

### Eligibility criteria

Eligible participants were non-smokers with good age-appropriate health conditions, as established by medical history, physical examination, and results of biochemistry, hematology and urine analysis testing four weeks prior to the study (see section *Laboratory tests*). Eligible participants required normal blood pressure and pulse rate according to reference normal ranges.

The sample excluded any participants who had been subjected to known enzyme-inducers/inhibitors within 30 days prior to the start of the study, and participants who took any medication less than two weeks prior to the trial starting date. Participants susceptible to allergic reactions to empagliflozin, or any other condition that might interfere with drug absorption, including prior surgery of the gastrointestinal tract, gastrointestinal diseases were excluded. Those with renal diseases, cardiovascular diseases, hepatic diseases, hematological disease or pulmonary disease, having dehydration, hypotension, urinary tract infections and cases of abnormal laboratory values were rejected. Finally, participants who had donated blood or who had been in multiple dosing study requiring a large volume of blood (more than 500 ml) to be drawn within six weeks preceding the start of the study were also excluded.

### Laboratory tests

Screening assessments encompassed complete (past and present) medical history evaluation, physical examination and laboratory tests, namely: (1) biochemical tests of fasting blood sugar, serum urea, serum creatinine, serum glutamic-pyruvic transaminase, serum glutamic-oxaloacetic transaminase, cholesterol, triglycerides; high-density lipoprotein, low-density lipoprotein, hepatitis c virus antibody, human immunodeficiency virus, blood Group (A, B, AB, O) and RH-typing; (2) a complete blood count report; (3) a urine analysis report, which included physical examination, chemical examination and microscopical examination.

### Intervention

The participants were allowed to eat a standard meal containing carbohydrates, protein and fats according to the allowed ratios of standard calories per day (2000 kcal/day).

The participants entered the Drug Research Center one day before the treatment began, in which they received their morning or evening treatment doses by a resident physician and a clinical pharmacist.

Each participant had to go to another room to receive the dose and then come back to the main room. Evening dose was given on the 2 ^nd^ of October and morning dose was on the 10 ^th^ of October 2018.

In order to increase patient compliance during the trial, incentives were offered, free meals (as above) and transportation after the end of the trial.

 All participants received their evening does first, stayed for 24 hours to have their urine and blood tests; then came back 7 days later to receive their morning dose, and stayed for 24 hours for urine and bloods, then came back for a follow-up at 48 hours.

Eligible healthy participants received a single oral dose of empagliflozin (JARDIANCE ®; 10 mg film coated tablet; manufactured by Boehringer Ingelheim Pharma GmbH & Co. KG, Germany for Boehringer Ingelheim International GmbH, German) in the evening or morning ^17^.

In the evening phase, at around 7 pm, the participants were administered the evening oral dose followed by dinner. After the washout period (7 days), at around 9 am, the participants then received the morning oral dose followed by breakfast.

The trial medication was administered with a full glass of water. The participants were asked to fast for at least 12 hours before the dose of empagliflozin (JARDIANCE ® 10 mg) and the dose was administered under close supervision of a medical investigator.

### Endpoints

Primary pharmacokinetic endpoint was to determine the effect of morning versus evening doses on pharmacokinetics parameters: T _max_ (h), C _max_ (ng/ml), AUC _0-t_ (ng.h/ml), while secondary pharmacokinetics parameter was AUC ( _0 to ∞_) (ng.h/ml) ^18^.

Pharmacodynamic endpoint was to determine the cumulative UGE (cumulative amount of glucose excreted) over the 24 h in g/dl ^18^.

### Pharmacodynamics evaluation

Urine samples were collected by urine collecting tube (1liter) for determination of UGE in g (urinary glucose excretion), which is the mean amount of glucose excreted in urine over the first 24 h after oral administration. Sampling intervals were 0 to 4, 4 to 8, 8 to 12 and 12 to 24 h after the administration of empagliflozin at the morning and evening doses.

Urine samples were stored at -80 °C before being sent to the clinical laboratory for analysis. Analysis was performed by the glucose hexokinase enzymatic method for determination of glucose concentration ^19^.

Cumulative UGE (mg) was calculated for each participant by multiplying the urine volume by the glucose concentration for each sampling interval in each period (morning or evening).

### Pharmacokinetics evaluation and bioanalysis

Serial blood samples (0.5 ml) for determination of plasma empagliflozin concentrations were collected at 0 h pre-dose and then at 0.333, 0.667, 1, 1.25, 1.5, 1.75, 2, 2.5, 3, 4, 6, 8, 10, 12, 24, and 48 h for period one (evening dose) and period two (morning dose). Bioanalytical method validation was done by the authors; blood samples treated with EDTA were centrifuged at 3500rpm for 5 minutes to get a plasma supernatant.

Plasma samples were analyzed by high performance liquid chromatography tandem mass spectrometry (HPLC- MS/MS) using an LC Agilent 1200 series and an Agilent 6410 Quadrupole mass spectrometer (Agilent Technologies, Inc., Santa Clara, CA) for determination of empagliflozin concentration in plasma. For preparation of plasma samples, 50ul of the internal standard dapagliflozin was added to an aliquot of 0.5ml plasma then vortex mixing was applied for 30 seconds. A liquid-liquid extraction by 2mls of diethyl ether-dichloromethane 60:40v/v was performed. After vortex mixing samples with the organic solvent for 2 minutes, samples were prone to phase separation at 3500rpm for 5 minutes in the centrifuge (Hermle Z 326K, Hermle Labortechnik GmbH, Wehingen, Germany). The clear supernatant was separated after centrifugation and evaporated under vacuum in the concentrator at 45 ^0^C (Vacufuge® Plus, Eppendorf, Germany). Reconstitution of dry residue by the mobile phase (0.4% formic acid:Acetonitrile 17:83v/v) was performed prior to injection on LC/MS/MS. An aliquot of 5ul was injected on an isocratic system with a mobile phase composed of 0.4% formic acid: Acetonitrile 17:83% v/v and a C18 column as the stationary phase (Gemini C18 50 X 4.6mm, particle size 5um, Phenomenex Inc, Torrance, CA.) with a total run time of 3.1 minutes. Empagliflozin and dapagliflozin were determined by mass spectrometry in the multiple reaction monitoring mode (MRM). The mass to charge ratios (m/z) of precursor ions monitored for empagliflozin and dapagliflozin were 429.4 and 423.4 respectively with a common product ion m/z 207.1. The method of analysis showed a linear calibration range from 0.5 to 200 ng/ml with a correlation coefficient (r2=0.999) and average accuracy percent of 98.7%. Inter-day precision for quality control samples were below a percent relative standard deviation of 5.5% confirming a precision within the acceptable limits of validation.

Pharmacokinetics parameters of Empagliflozin were analyzed by Winnonlin™ software version 2.0 (Pharsight, California, Palo Alto, CA). C _max_, T _max _were calculated directly from the plasma concentration time curve (shown in Results section), while AUC _0-t_ and AUC _0 to ∞. _ were computed using the linear trapezoidal rule.

### Safety and tolerability assessment

The subjects were followed up during and after one day of the study. Results obtained from the laboratory tests (biochemical, complete blood picture report, and urine analysis report) were done prior to the study with only participants with results within the normal range allowed to participate in the study. In addition, medical history and physical examination were done and evaluated by a physician in the research center prior to the start of the study. Adverse effects were recorded by participants during the two periods (night and day).

### Statistical analysis

Based on a within subject variability of 18.7% for C _max _of empagliflozin ^20^, a calculated sample size of 16 subjects was sufficient to acquire a study power of 80% at a level of significance of 5% ^21, 22^.

All subjects were tested for the pharmacokinetics and pharmacodynamics endpoints; analysis of the study was intention to treat. Diurnal and nocturnal variability from morning and evening doses were assessed independently for all the pharmacokinetics and pharmacodynamics parameters.

Pharmacokinetics primary endpoints (C _max_, AUC _0-t_) and secondary endpoint (AUC _0-∞_) were tabulated; one-way analysis of variance (ANOVA) was used, the data was assessed as mean difference between the two phases. Results were shown as P-value, geometric mean with 90% CI (confidence interval). Pairs of logarithmic transformed data of the primary and the secondary of the endpoints were analyzed using SAS ^® ^University edition statistics software (SAS ^®^, USA) to determine a statistical difference between the morning and evening phases. T _max_ was analyzed by Pairwise comparisons using the Wilcoxon signed-rank test. Median and range for both morning and night data and difference in median and P value were reported.

Pharmacodynamics parameter (cumulative amount of glucose excreted) was shown as P-value, geometric mean with 90% CI. Assessment was done by comparing mean difference of cumulative amounts of urine excreted over the 24 h for each phase (morning or evening) by one-way analysis of variance (ANOVA) for determining a statistical difference between the morning and evening phases using SAS ^® ^University edition statistics software. P-value of < 0.05 and 90% CI for a two-tailed test were used to assess the significant difference in the study hypothesis.

## Results

### Participant demographics

In total, 16 adult men participated in the study; median age was 26 years (range 18–55 years) with median body mass index of 24.285 kg/m ^2^ (range 20–30 kg/m ^2^). The baseline demographics of all study participants are shown in Table 1. All 16 participants completed the study (no drop out), and all results were included in the pharmacokinetics, pharmacodynamics.

**Table 1.** Baseline demographic and clinical characteristics of the 16 enrolled participants in the study.

Variable, mean (SD)		Phase 1 (n=16)	Phase 2 (n=16)
Age, years		30.5 (12.4)	30.5 (12.4)
Weight, Kg		74.5 (10.4)	74.5 (10.4)
BMI, Kg/m ^2^		24.9 (3.1)	24.9 (3.1)
Blood pressure, mmHg	Systole	114.5 (5)	111.5 (3.5)
	Diastole	74 (5)	71.9 (4)
Fasting blood sugar (mg/dl)		84.4 (10.19)	84.4 (10.19)
Triglycerides (mg/dl)		118.5 (33.25)	118.5 (33.25)
HDL (mg/dl)		49.375 (9.07)	49.375 (9.07)
LDL (mg/dl)		71.6125 (20.6)	71.6125 (20.6)

### Pharmacodynamics

**Figure 2.**
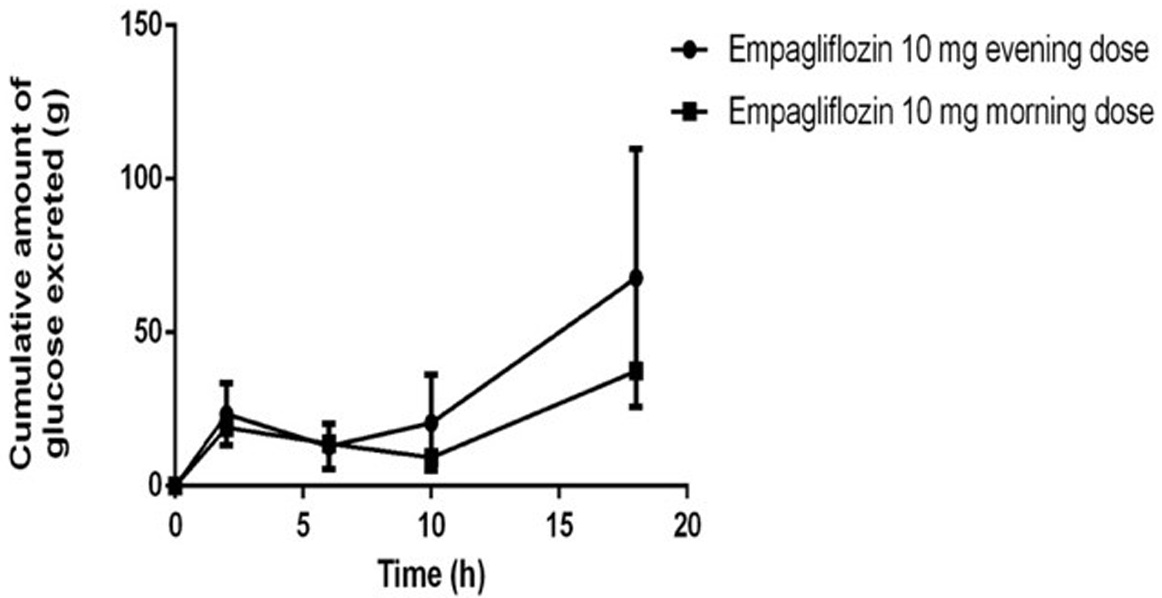
Cumulative amount of glucose excreted for the evening (phase 1) and morning (phase 2) after administration of empagliflozin 10 mg.

**Figure 3.**
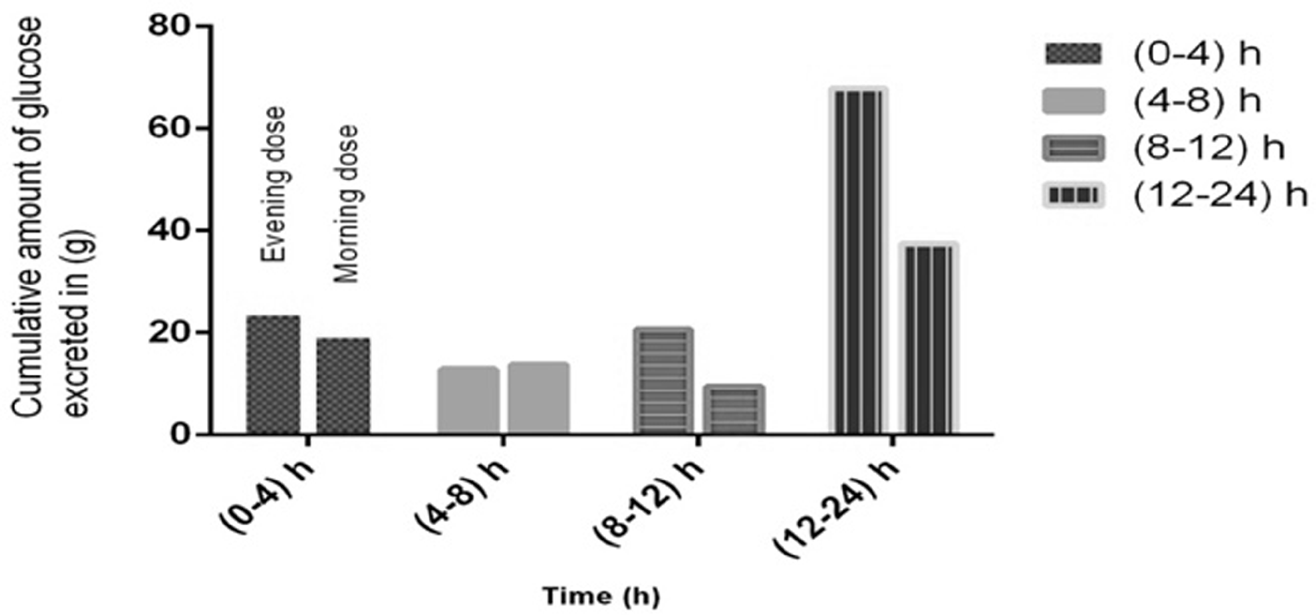
Cumulative amount of glucose excreted over the 24 h for phase 1 and phase 2 after administration of single oral dose of empagliflozin 10 mg.

Mean cumulative amount of glucose excreted from a single dose of empagliflozin (10 mg) over the 24 hours in the evening (Phase 1) and morning (Phase 2) doses are shown in Table 2 ( Figure 2 and Figure 3). Mean UGE (0–24) for the evening dose (phase 2) was 69 (CV%=43.4), which was higher compared to the morning (phase 1), at 39 (CV%=41). Geometric mean results comparing the evening and morning doses for the log transformed UGE values was 116.7 (90% Cl 79.8-170.8; P=0.7317).

**Table 2.** Cumulative amount of glucose excreted after single dose administration of empagliflozin 10 mg.

Parameter, mean±SD	Time intervals	Phase 1 (n=16)	Phase 2 (n=16)
Cumulative amount of glucose excreted (UGE) (g/dl)	(0-4) h	23.5±10	19±22
	(4-8) h	13±7	14±10
	(8-12) h	21±16	9±6
	(12-24) h	69±30	39±16

### Pharmacokinetics

**Figure 4.**
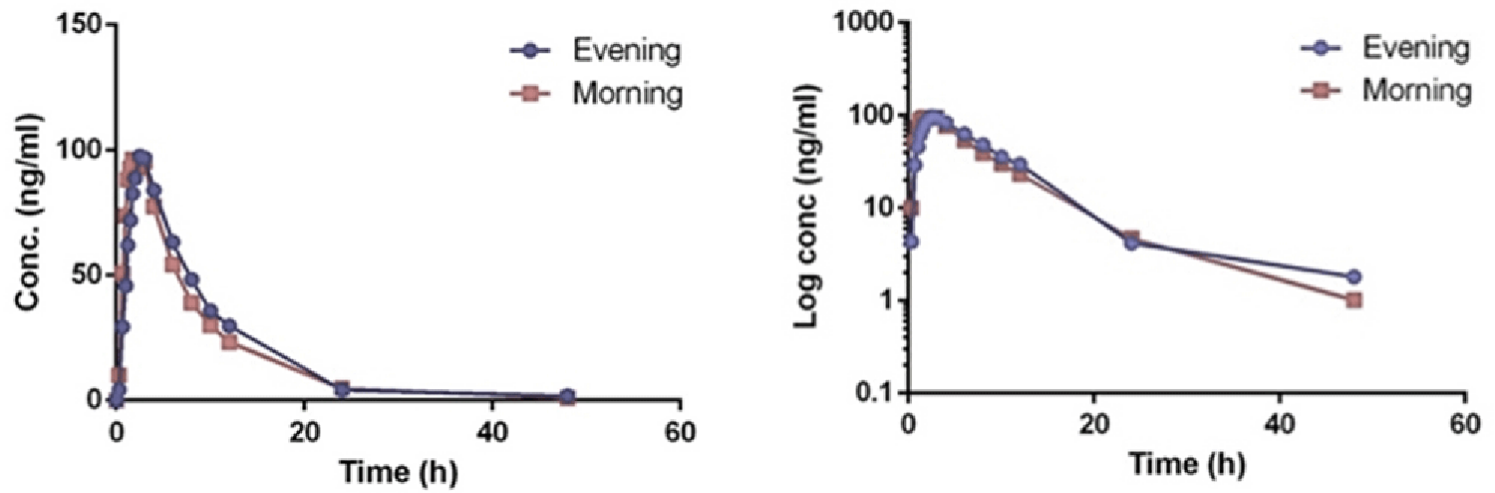
Linear (left panel) and semi-logarithmic plot (right panel) for average empagliflozin 10 mg plasma concentration (ng/ml) after evening (phase 1) and morning (phase 2) doses.

Evening and morning doses of empagliflozin once daily were rapidly absorbed (in favor the morning dose) with peak median T _max_ ranged between 2.5 (interquartile range (IQR) 1.25) to 1.625 (IQR 1.3) h post dose (P=0.063), reaching the maximum concentration between 125.60±30.7 (24.4%) to 117.9±32 (27.16%) g (P=0.324). The latter was followed by the slow elimination phase with elimination half-lives in the evening and morning doses of 7.2 (IQR 1.6) to 7 (IQR1.4) h, respectively ( Table 3, Figure 4).

**Table 3.** Pharmacokinetics findings of empagliflozin 10 mg.

Parameter	Phase 1 (n=16)	Phase 2 (n=16)	P-value
T _max_, h, median (interquartile range)	2.5 (1.25)	1.625 (1.3)	0.063
C _max_, ng/ml, mean (SD)	118 (32)	125.6 (30.7)	0.324
AUC _0-t_, ng.h/ml, mean (SD)	960 (157)	888 (187)	0.057
AUC _0-∞_, ng.h/ml, mean (SD)	980 (161.6)	899 (189)	0.036

In addition, the total exposure of the drug in a form of the area under the curve (AUC _0-t_) between the morning and evening doses ranged from 888 (21.05%) to 960 (16.35%) (P=0.057). AUC _0-∞_ ranged between 899 (21.02%) to 980 (16.4%) (P=0.036) ( Table 3, Figure 4). All the findings above suggests a linear pharmacokinetics relationship with time.

In addition, the geometric mean results of comparing log transformed C _max_, AUC _0-T_ and AUC _0-∞_ for the evening and morning doses were 93.6 (90% Cl 83.707-104.82), 108.9 (90% Cl 101.274-117.297) and 109.823, (90% Cl 102.243-117.964), respectively.

The total exposure of the drug measured by AUC _0-t_ relative to the extrapolated total AUC _0-∞_ of empagliflozin was slightly higher for evening than morning doses (98.776±0.541 *vs* 98.030±0.794).

### Safety analysis

No adverse events were detected from empagliflozin 10 mg in this study. The drug was well tolerated for all participants. Vital signs before, during and after the end of the study (blood pressure, pulse) were within normal ranges. Clinical and lab values were all evaluated to be within normal values.

## Discussion

Chronopathology proposes that any disease, including DM, may occur as a result of disruption of biorhythms ^23^. The effect of circadian clock on the kidney may affect the pharmacokinetics and/or pharmacodynamics of many drugs, especially those that exert their mechanism of action in the kidney ^7^. Moreover, circadian clock proteins control most hormones, enzymes, and transport mechanisms related to glucose metabolism ^24^. The following examples show different techniques on how circadian rhythm influence the kidney’s action: it has been described that renal function cycles over the 24 h and follows rhythmicity in its action, of note, GFR is influenced by many factors such as; systemic blood pressure, renal blood flow, afferent and efferent arteriolar resistance regulation, sympathetic system and hormones as renin and vasopressin ^25^.

Factors affecting GFR also affect other mechanisms within the kidney. Stimulation of the sympathetic nervous system influences renin secretion and renal sodium reabsorption (which can affect the action of our study drug) ^25^. Previous studies showed that SGLT2 expression in the proximal tubules is increased in DMT2, which may suggest that further studies to be done on the expression of SGLT1 and SGLT2 at the proximal tubules in the kidney (in which empagliflozin inhibit) are required ^14^.

Our study examined the effect of the SGLT2i with empagliflozin, taking into account that the transport of solutes in and out of the kidney are controlled by the circadian clock. It has been shown that NH3 and SGLT1 are controlled by circadian oscillations proteins (Per 1 and BMA1), affecting the mRNA transcription ^26^.

Other studies determined the mechanism and the level of involvement of the circadian clock genes and circadian rhythms in the proximal tubule cellular Na+/H+ ex- changer 3 (NH3) transporter. In addition to other mutations in circadian clock proteins, rhythmic oscillations involved in NH3 activity are directly related to positive daily variations in sodium and water transport of the proximal cells ^27^. NH3 expression is activated in the dark cycle and increases during a food intake ^26, 28^.

As sodium-hydrogen exchanger is affected by the circadian clock and, due to the link between empagliflozin (SGLT2i) and sodium-hydrogen exchanger, contributes to preventing heart failure. We recommend performing further studies on the effect of circadian rhythm on the action of SGLT2 inhibitors in specific and antihyperglycemic agents in general ^29^.

Circadian clock genes appear to be involved in every biological process in the human body. Currently, there is a lack of studies examining and determining the effect of chronopharmcology on antihyperglycemic drugs and drugs that exert their activity on the kidney. We recommend more chronopharmacological studies for antihyperglycemic drugs, either working on insulin secretion level or, as per our study, or drugs, which exert their mechanism of action on the kidney ^5, 24^.

A few recent studies have started to address the relationship between chronopharmacology and antihyperglycemic drugs. One recent study examined the effect of administrating dapagliflozin on high-fat diet-induced obesity in mice; the results of the study were promising and suggest that dapagliflozin follows chronopharmacology as plasma glucose, insulin levels and adipose adipokines decreased in the light phase ^30^.

Empagliflozin (JARDIANCE®) is available in two doses: 10 and 25 mg ^31^. We examined the 10 mg dose. Empagliflozin can be taken once daily (without rationale on its timing) as oral bioavailability of empagliflozin is high (higher *t*_½_) ^2^. In the current study, empagliflozin 10 mg once daily in the morning or evening was rapidly absorbed, reaching the peak t _max_ (C _max_) range between 2.5 and 1.625 h post dose, which similar to other previous studies on diabetic patients and healthy volunteers (1.5 to 2.1 h) ^2, 32, 33^. Absorption followed by the slow elimination phase with approximately the same elimination half-life in the evening and morning doses (7.278–7.327 h), which appeared shorter compared to previous studies that reached up to 13 h ^2, 18^.

The results of the current study showed that the total exposure of the drug measured by AUC _0-t_ relative to the extrapolated total AUC _0-∞_ of empagliflozin was slightly higher for evening than morning doses (98.776±0.541, 98.030±0.794). On the other hand, UGE (0–24) for the evening dose was 69 g, compared to the morning phase, which was quite low at 39 g. Comparing these results to previous studies examining once daily dose of empagliflozin 10 mg, UGE _0–24_ was 47.9 in healthy Caucasian and 50.6 g in healthy Japanese populations, respectively ^21, 34^. Moreover, in diabetic patients it ranged between 46.3 and 89.8 for the single dose to all doses of empagliflozin ^2^.

In studies done on diabetic patients, UGE _0–24_ for the single dose of 10 mg ranged between 74.9 g and 77.9 g ^32, 33^. In this study, although the UGE _0–24_ for the evening dose was higher, it didn’t reach a statistically significant level ^35^.

Moreover, the study on healthy Japanese participants showed that as the exposure increased (C _max_ and AUC _0-∞_), the UGE increased, which might correlate with our study findings (higher AUC _0-∞_, UGE _0–24_) favoring the night dose, except that C _max_ didn’t differ between morning and evening doses ^21^.

The reason behind the higher UGE (0–24) for the evening dose may be attributed to the short duration of the study or may result from the effect of food since the dinner meal is always higher in calories, compared to the morning meal. However, previous studies that examined the role of food on empagliflozin (50 mg) showed that UGE _0–24_ didn’t differ significantly between the fasted and fed state, with 71.7 g (13.6) and 75.9 g (17.9) mean (SD) respectively in healthy volunteers ^34^. Moreover, another study, done on the 25 mg dose, showed a non-significant effect with food administration (geometric mean 84.04, 90% CI 80.86-87.34) for the AUC _0-∞._^36^. As a result, due to the limited studies on the effect of food on the morning and evening doses from the pharmacokinetics and pharmacodynamics perspective, further studies may be required.

Due to the above-mentioned functions of empagliflozin, we studied the effect of circadian rhythm and its time of administration regarding its pharmacokinetics and pharmacodynamics parameters, as we aim to achieve the best use of the drug, towards implementing personalized medicine.

This study was a pilot, done on healthy participants to limit confounders (other drugs or commodities) that can affect the study aim. Other studies performed on DM patients (their characteristics and physiological effects) are recommended to further explain if circadian rhythm can affect the pharmacokinetics and pharmacodynamics of the drug and thus efficacy of antihyperglycemic drugs. In addition, more studies can be performed addressing the role of pharmacogenetics changes and chronopharmacology.

## Conclusion

Although there was a difference in the overall exposure of the empagliflozin in the morning vs evening doses (P value was significant for AUC _0-t _and AUC _0-∞_), they all were within the bioequivalence range. The difference in the other empagliflozin pharmacokinetic parameters between the evening and morning doses were non-significant. In addition, (UGE _0–24_) was higher for the evening dose, but it didn’t reach a significant level. Taken together, the findings of the current study provide the first evidence that there is non-significant difference in the pharmacokinetics and pharmacodynamics effects between evening and morning dosing of empagliflozin 10 mf film-coated tablets.

## Data availability

### Underlying data

Dryad: Demographic data, https://doi.org/10.5061/dryad.gqnk98smc ^37^.

This project contains the following underlying data:

- Data file 1: (Demographic data of the participant at before the start of the Evening dose)
- Data file 2: (Demographic data of the participant at before the start of the Morning dose)

Dryad: Pharmacodynamics parameters, https://doi.org/10.5061/dryad.k0p2ngf7b ^38^.

This project contains the following underlying data:

- Data file 1 (Cumulative amount of glucose excreted over the 24 hours at the Morning dose)
- Data file 2 (Cumulative amount of glucose excreted over the 24 hours at the Evening dose)
- Data file 3 (Pharmacodynamics evaluation Urinary glucose secretion)
- Data file 4 (Pharmacodynamics parameters of Morning and Evening doses of Empagliflozin)

Dryad: Pharmacokinetics parameters, https://doi.org/10.5061/dryad.brv15dv8j ^39^.

This project contains the following underlying data:

- Data file 1 (Plasma concentration(s) levels of the 16 participants at the Evening dose)
- Data file 2 (Plasma concentration(s) levels of the 16 participants at the Morning dose)
- Data file 3 (Pharmacokinetics parameters of the 16 participants at the Evening dose)
- Data file 4 (Pharmacokinetics parameters of the 16 participants at the Morning dose)
- Data file 5 (AUC0-t and AUC0-inf of the 16 participants for both evening and morning doses).

### Extended data

Dryad: Bioanalytical method validation, https://doi.org/10.5061/dryad.brv15dv8j ^39^.

Dryad: Protocol, case report(s), ethics approvals, inclusion and exclusion criteria, https://doi.org/10.5061/dryad.7h44j0zt1 ^40^.

This project contains the following underlying data:

- Data file 1: (The trial protocol)
- Data file 2: (Empty form of case report) this file is not at https://doi.org/10.5061/dryad.7h44j0zt1
- Data file 4: (Inclusion and Exclusion criteria for the enrollment of the participants in the trial)
- Data file 5: (Ethics committee approval(s) of AinShms University)

### Reporting guidelines

Dryad: TREND checklist for ‘The effect of morning versus evening administration of empagliflozin on its pharmacokinetics and pharmacodynamics characteristics in healthy adults: a two-way crossover non-randomised trial’, https://doi.org/10.5061/dryad.gqnk98smc ^37^.

Data is licensed under a CC0 1.0 Universal (CC0 1.0) Public Domain Dedication license.
