# Supplementary material for: The effect of morning versus evening administration of empagliflozin on its pharmacokinetics and pharmacodynamics characteristics in healthy adults: a two-way crossover, non-randomised trial
Source: F1000Res. 2021 Apr 26;10:321. [Version 1] doi: 10.12688/f1000research.51114.1 (PMC8167502; doi:10.12688/f1000research.51114.1)
Supplement: Peer review history

# Peer review: Reviewer response for version 1

**DOI:** 10.5256/f1000research.54231.r85394

**Published:** 2021-05-28

**Authors:** Samar Darwish

**Affiliations:** 1 Pharmacology and Toxicology Department, Badr University, Cairo, Egypt

In this manuscript, the authors investigated the chronopharmacological effects of morning vs evening empagliflozin administration (10 mg) to healthy Egyptian adults, by studying the pharmacokinetics and some pharmacodynamics characters of the drug. The study sheds the light on the possibility that circadian rhythm can affect drug properties, and may inspire other researchers to study this theory in diabetic patients. 

 However, there are few minor revisions that I advise the authors to take in concern:

1. Abstract section:
  1. In Methods part: please add “in” before “the morning” word to be more clear for the readers.
  2. In Methods part: please mention “The urine glucose excreted” before its abbreviation “UGE” and remove “glucose excreted” from the Results part.
  3. In Conclusion part: would you please mention the P value used for the statistical findings, for it will help other researchers for further studies.
2. Introduction section:
  1.   Please rephrase the sentence “the blood pressure lowering drug Fimasartan shows pronounced blood pressure lowering (readjusting the dipping pattern) at night compared to morning dose.” In the 9 ^th^ paragraph to be more clear.
3. Methods section:
  1. Please also mention the number of participants in the text as you mentioned in the figure.
  2. In the Eligibility criteria part: you have mentioned that the study excluded some participants, while in the study design figure the excluded number was zero. Please check.
  3. In the Endpoints part: adjust the abbreviation meaning of UGT as requested before.
4. Results section:
  1. In table 1: Why did you express the data with median instead of the mean??
  2. What is the difference between the data represented in figure 1 and figure 2? If there, please clarify.
  3. If applicable, I recommend mentioning the statistical significance or insignificance when comparing the results of evening dose to the morning dose all over the result section.
5. Discussion section:
  1. In the first paragraph: the sentence starting with “The following examples show…..” is too long, please try to rephrase it.
  2.  In the second paragraph: I’d prefer to replace the sentence “which may suggest that further studies to be done on the expression of SGLT1 and SGLT2 at the proximal tubules in the kidney (in which empagliflozin inhibit) are required” with the following sentence “which may suggest that further studies are required on the inhibitory site of empagliflozin, which is the expression of SGLT1 and SGLT2 at the proximal tubules”
  3. In the 3 ^rd^ paragraph: clarify what did “i” refer to in the abbreviation “SGLT2i”
  4. Also in the 3 ^rd^ paragraph: “Na+/H+ ex- changer 3” should be mentioned before its abbreviation “NH3”, and removed from the 4 ^th^ paragraph
  5. It would be better to rephrase paragraph number 5. 

Is the work clearly and accurately presented and does it cite the current literature?

Yes

If applicable, is the statistical analysis and its interpretation appropriate?

Yes

Are all the source data underlying the results available to ensure full reproducibility?

Yes

Is the study design appropriate and is the work technically sound?

Yes

Are the conclusions drawn adequately supported by the results?

Yes

Are sufficient details of methods and analysis provided to allow replication by others?

Yes

Reviewer Expertise:

pharmacokinetics, pharmacodynamics, endocrine disorders, Drug discovery, inflammatory diseases, neurological disorders, single-cell technologies.

I confirm that I have read this submission and believe that I have an appropriate level of expertise to confirm that it is of an acceptable scientific standard.

# Peer review: Reviewer response for version 1

**DOI:** 10.5256/f1000research.54231.r85636

**Published:** 2021-05-28

**Authors:** Ashraf Abdel-Naim

**Affiliations:** 1 Department of Pharmacology and Toxicology, Faculty of Pharmacy, King Abdulaziz University, Jeddah, Saudi Arabia

The effect of morning versus evening administration of empagliflozin on its pharmacokinetics and pharmacodynamics characteristics in healthy adults: a two-way crossover, non-randomized trial.

- Comment 1: the manuscript is well-written, and the study hypothesis is clear, although; more information about previous studies with similar ideas should be addressed in detail and specifically focusing on the number of patients enrolled on those similar studies
- Comment 2: I believe the manuscript will benefit from limitation section that address all the concerns from the study
- Comment 3: I find it really interesting that information in Table 1 are almost the same after a 7-day washout period. Readings such as fasting blood glucose, Triglycerides, HDL, LDL can be changed very dramatically in one day.

**Minor Comments:**

- In the abstract section: in the methods part, line 9 ‘was done prior to injection in LC/MS/MS’ please rephrase this sentence.
- In the intervention section, line 13: please rephrase this sentence ‘in the evening or morning’.
- In the intervention section, last line ‘dose was administered under close supervision of a medical investigator’ should be added with line 4.
- In Endpoint section: ‘pharmacokinetic endpoint was to determine’ should be ‘pharmacokinetic endpoints were to determine’.
- In discussion section: paragraph 3‘Our study examined the effect of the SGLT2i with empagliflozin, taking into account that the transport of solutes in and out of the kidney are controlled by the circadian clock. It has been shown that NH3 and SGLT1 are controlled by circadian oscillations proteins (Per 1 and BMA1), affecting the mRNA transcription’ need to be clearer.

**I suggest approval with minor revision.**

Is the work clearly and accurately presented and does it cite the current literature?

Yes

If applicable, is the statistical analysis and its interpretation appropriate?

Yes

Are all the source data underlying the results available to ensure full reproducibility?

Yes

Is the study design appropriate and is the work technically sound?

Yes

Are the conclusions drawn adequately supported by the results?

Yes

Are sufficient details of methods and analysis provided to allow replication by others?

Yes

Reviewer Expertise:

NA

I confirm that I have read this submission and believe that I have an appropriate level of expertise to confirm that it is of an acceptable scientific standard, however I have significant reservations, as outlined above.

# Peer review: Reviewer response for version 1

**DOI:** 10.5256/f1000research.54231.r84059

**Published:** 2021-05-12

**Authors:** Hesham Basma

**Affiliations:** 1 University of Nebraska Medical Center, Omaha, NE, USA

Herein, Rana M. ElDash and colleagues report findings on the pharmacokinetics and pharmacodynamics characteristics of morning versus evening administration of empagliflozin in healthy adults. The authors concluded that there is no statistical significance between the morning and evening doses of empagliflozin. The findings are very timely and would be well received. However, there are few minor revisions that should be addressed by the authors.

1. In the abstract, methods section, please rephrase the following sentence to be clearer, “in the evening, and after a seven-day washout period, the morning”
2. Several studies have been done for Statins chronotherapy. It is very relevant to the current study. I would encourage the authors to include a reference for it in the introduction.
3. In table one, the same individuals participated in both phases and it is redundant to mention the same numbers in phases one and two, I would suggest making it one column (with parameter in the header instead of phase1 and phase2).
4. Is it possible to include Standard Error (S.E.) in figure 3?

Is the work clearly and accurately presented and does it cite the current literature?

Yes

If applicable, is the statistical analysis and its interpretation appropriate?

Yes

Are all the source data underlying the results available to ensure full reproducibility?

Yes

Is the study design appropriate and is the work technically sound?

Yes

Are the conclusions drawn adequately supported by the results?

Yes

Are sufficient details of methods and analysis provided to allow replication by others?

Yes

Reviewer Expertise:

Molecular and cell biology

I confirm that I have read this submission and believe that I have an appropriate level of expertise to confirm that it is of an acceptable scientific standard.
